# Delineating the Ultra-Low Misorientation between the Dislocation Cellular Structures in Additively Manufactured 316L Stainless Steel

**DOI:** 10.3390/ma17081851

**Published:** 2024-04-17

**Authors:** Fei Sun, Yoshitaka Adachi, Kazuhisa Sato, Takuya Ishimoto, Takayoshi Nakano, Yuichiro Koizumi

**Affiliations:** 1 Department of Material Design Innovation Engineering, Nagoya University, Furo-cho, Chikusa-ku, Nagoya 464-8603, Japan; 2 Research Center for Ultra-High Voltage Electron Microscopy, Osaka University, 7-1 Mihogaoka, Ibaraki 567-0047, Japan; sato@uhvem.osaka-u.ac.jp; 3 Aluminium Research Center, University of Toyama, 3190, Gofuku, Toyama 930-8555, Japan; ishimoto@sus.u-toyama.ac.jp; 4 Division of Materials and Manufacturing Science, Graduate School of Engineering, Osaka University, 2-1 Yamadaoka, Suita 565-0871, Japan; nakano@mat.eng.osaka-u.ac.jp (T.N.); ykoizumi@mat.eng.osaka-u.ac.jp (Y.K.); 5 Anisotropic Design and Additive Manufacturing Research Center, Osaka University, 2-1 Yamadaoka, Suita 565-0871, Japan

**Keywords:** additive manufacturing, cellular structure, misorientation, transmission electron microscopy, transmission Kikuchi diffraction, electron backscatter diffraction

## Abstract

Sub-micro dislocation cellular structures formed during rapid solidification break the strength–ductility trade-off in laser powder bed fusion (LPBF)-processed 316L stainless steel through high-density dislocations and segregated elements or precipitates at the cellular boundaries. The high-density dislocation entangled at the cellular boundary accommodates solidification strains among the cellular structures and cooling stresses through elastoplastic deformation. Columnar grains with cellular structures typically form along the direction of thermal flux. However, the ultra-low misorientations between the adjacent cellular structures and their interactions with the cellular boundary formation remain unclear. In this study, we revealed the ultra-low misorientations between the cellular structures in LPBF-processed 316L stainless steel using conventional electron backscatter diffraction (EBSD), transmission Kikuchi diffraction (TKD), and transmission electron microscopy (TEM). The conventional EBSD and TKD analysis results could provide misorientation angles smaller than 2°, while the resolution mainly depends on the specimen quality and scanning step size, and so on. A TEM technique with higher spatial resolution provides accurate information between adjacent dislocation cells with misorientation angles smaller than 1°. This study presents evidence that the TEM method is the better and more precise analytical method for the misorientation measurement of the cellular structures and provides insights into measuring the small misorientation angles between adjacent dislocation cells and nanograins in nanostructured metals and alloys with ultrafine-grained microstructures.

## 1. Introduction

The intentional manipulation of dislocations through conventional strengthening strategies like grain refinement, solid solution strengthening, and precipitation strengthening plays a pivotal role in tailoring metallic materials for superior performance by effectively introducing extrinsic obstacles to dislocation motion during plastic deformation [1,2]. A dislocation cell is a typical dislocation structure in heavily deformed metallic materials. Its generation and multiplication result from the strain energy reduction in glide dislocations and are associated with the self-organization of discrete dislocations that have become mutually trapped [3]. One of the requirements for dislocation cell formation is that dislocations have sufficient mobility out of their slip plane. During the dislocation cell-forming process, the lattice dislocations re-arrange to minimize the total energy state, forming dense dislocation walls, which act as obstacles to effectively hinder the free movement of the dislocations, leading to increased resistance to deformation [4,5,6,7]. The dislocation cells are confined by low-angle grain boundaries [8]. The size of the dislocation cells ranges from several micrometers to the sub-micrometer regime, with size being proportional to the applied stress or strain [9].

However, to date, a precise depiction of the underlying mechanisms of dislocation cells remains unrealized in additive manufacturing (AM)-processed metallic materials, which possess various solidification microstructure features spanning a wide range of length scales, involving dislocation cellular structures and a high density of dislocations at an as-built state without plastic deformation. This phenomenon primarily arises from the complex thermal history, enabling large solidification temperature gradients and ultra-high cooling rates during processing. The typical features of dislocation cellular structures involve major high-density cellular boundaries formed by tangled dislocations and minor statistically stored dislocations or dislocation-free in some cases in the cellular interiors. The average dislocation cell size at the cross-sections of the dislocation cellular structures ranges from 300 nm to 1 μm, depending on the laser processing parameters. Many metallic materials processed via AM exhibit ultrafine dislocation cellular structures, leading to extraordinary mechanical properties [10,11,12]. In some cases, these materials break the strength–ductility trade-off achieved by the cellular structure with high-density dislocations and solute micro-segregation or precipitate at the cellular boundaries. This achievement represents a significant advance not easily accessible through traditional processing methods but as a peculiar feature that exists widely in additively manufactured metallic materials [10,11,12,13,14,15,16,17]. Note that these dislocation cellular structures are different from conventional dislocation cell walls despite the morphology similarities. In addition, during the cellular structure growth process, the dislocations move sufficiently and not limited in their slip systems but also out of their slip plane to balance the strain and energy among the surrounding cellular structures, which has been confirmed by the visible tangled dislocations with multiple Burgers vectors under various two-beam observation conditions using transmission electron microscopy (TEM) [17]. The various distributions of the cellular structure in the melt pool also mainly contribute to the different crystallographic textures of the AM-processed metallic materials [18,19,20]. Texture development during LPBF is initially dominated by epitaxial grain growth from grains of the substrate. This epitaxial solidification occurs through the cellular solidification features that develop from rapid directional solidification [21].

AM technologies for 316L stainless steel (SS) are of great interest due to their high efficiency, design freedom, and better mechanical properties than those manufactured by conventional methods. Laser powder bed fusion (LPBF) remains the most prevalent technique for metals and alloys compared with other printing methods. The dislocation cellular structure in LPBF-processed 316L SS tends to grow along the maximum temperature gradient from the melt pool boundary (MPB) toward the center of the melt pool and along the preferred growth direction of <001> in the face-centered cubic (FCC) structure [21]. Different laser scanning strategies also generate various distribution behaviors of cellular structures, thereby necessitating both imaging and quantitative measurements [10,22,23,24,25]. The spacing of the cellular structures can be calculated based on the thermal gradient and the speed of the liquidus isotherm [21]. As the typical features of the cellular structure, the cellular size and orientation are highly dependent on temperature and strain. The high-density dislocation entangled at the cellular boundary accommodates solidification strains among the cellular structures and cooling stresses through elastoplastic deformation. However, the misorientations between adjacent cellular structures, the thickness of the cell boundaries, and their interactions with the cellular boundary formation and dislocation density are still open questions to the AM community. 

To fully understand the cellular structure behaviors, there remains a question of how to quantitatively measure the misorientations between the sub-micro cellular structures. Unlike the traditional large-angle grain boundary, the misorientation across the cellular boundaries in LPBF 316L SS is usually very low, and the number of fractions is unavailable to be measured directly, especially when the ultra-low misorientation is smaller than 2° using conventional electron backscatter diffraction (EBSD); however, line profiles of the misorientation angle and kernel average misorientation (KAM) map measured by EBSD analysis can indicate a misorientation smaller than 2° [10,17,26,27]. Traditional techniques like EBSD associated with scanning electron microscopy (SEM) require a large interaction volume between the electrons from the beam and the atoms of the materials, thereby lacking spatial resolution and causing inaccurate correspondence between the misorientation and dislocation cell. To obtain higher spatial resolution, the transmission Kikuchi diffraction (TKD) technique [28,29] enables the analysis of the microstructure of nanocrystalline materials using an SEM equipped with a standard EBSD system and a special holder for TEM foil installation. The spatial resolution of TKD is higher than conventional EBSD. In addition, transmission electron microscopy (TEM) offers the necessary spatial resolution to observe the nano-sized dislocation cells and obtain the misorientation between the adjacent dislocation cells from the Kikuchi-line analysis. 

This study aims to quantitatively evaluate the ultra-low misorientations among the cellular structures in the 316L SS samples processed by different LPBF parameters using the conventional EBSD, TKD, and TEM techniques, providing fundamental and indispensable evidence for further investigation of cellular structure behaviors, like the interactions with local strains, dislocation behaviors, and small-scale mechanical properties in AM-processed metallic materials.

## 2. Experimental Procedure

**Figure 1:**
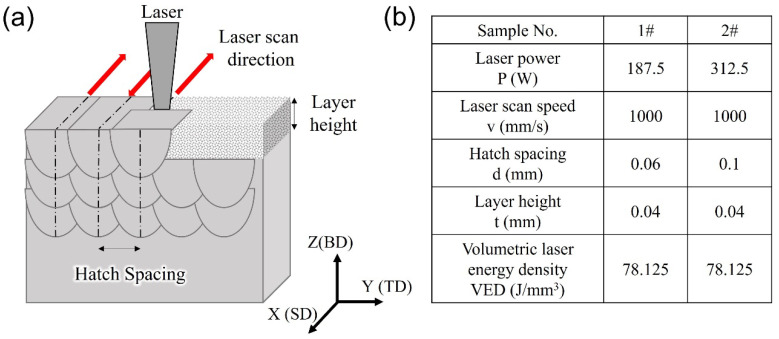
(**a**) Schematic diagram of the LPBF process; (**b**) LPBF parameters of the two samples.

The 316L SS spherical powders were manufactured by gas-atomization with a nominal composition of Fe-18Cr-14Ni-2.5Mo-0.03C (wt.%). The particle sizes were smaller than 53 μm. The 316L SS samples were printed as 10 × 10 × 10 mm cubes by the LPBF fabrication process using a 3D printer (EOSM290, EOS GmbH, Krailling, Germany) equipped with a Yb-fiber laser. The LPBF processing parameters are shown in Figure 1. Figure 1a displays the schematic diagram of the LPBF method making use of a laser beam energy source to melt and fuse powders in a layer-by-layer processing manner into a desired shape. The laser scan strategy, “X-scan strategy”, indicates the laser beam was scanned bidirectionally along the X-axis without rotation [30]. Figure 1b lists other LPBF processing parameters of the two samples in terms of laser powder (P), laser scan speed (v), hatch spacing (d), and layer height (t). The volumetric laser energy density (VED) was computed as VED = P/(vdt). The two 316L SS samples have the same VED but have different laser power and hatch spacing.

The specimens were cut from the as-printed cubic 316L SS samples to reveal the solidification microstructures of the cross-sections along the build direction (BD), laser scan direction (SD), and transverse direction (TD), then further polished using an automatic polishing machine (Mecatech 250 SPI, PRESI SAS, Eybens, France). The polished specimens were electrochemically etched in a solution of nitric and hydrochloric acid (HNO_3_:HCl:H_2_O = 1:10:10). The 3D microstructure can be visualized to understand the microstructure features, e.g., the dislocation cellular structures’ distribution behavior, which was similar to the one reported in our previous study [17]. The detailed characterization of dislocation cellular structure was carried out on the as-built specimens without heat treatment using field emission SEM (FE-SEM, JSM-7001F; JEOL Ltd., Tokyo, Japan) and TEM (JEM-2100PLUS; JEOL Ltd., Tokyo, Japan). According to the typical distribution of the cellular structures in the melt pool, the cross-section of the cellular structure was considered to make TEM specimens for better observation of the dislocation cells. TEM specimens were prepared using a focused ion beam (FIB) instrument (Scios2 Dual Beam; Thermo Fisher Scientific, Hillsboro, OR, USA) with gallium ions. Crystallographic orientation of the dislocation cellular structures was analyzed on bulk specimens using EBSD equipped in the FE-SEM. Orientation mapping of the TEM foil was performed using a high-performance focused ion beam system (FIB-SEM, MI-4000L; Hitachi High-Tech, Tokyo, Japan) by TKD technique. The inverse pole figure (IPF) map, kernel average misorientation (KAM) map, and misorientation angle analysis were subsequently analyzed based on the EBSD data using OIM TSL Analysis 7 software provided by EDAX. The misorientation angles between adjacent dislocation cells in TEM were evaluated based on the Kikuchi patterns of each dislocation cell, which were collected at the center of each dislocation cell first and then analyzed using the tools for orientation determination and crystallographic analysis (TOCA) v1.0 software package developed by S. Zaefferer [31]. 

## 3. Results and Discussion

**Figure 2:**
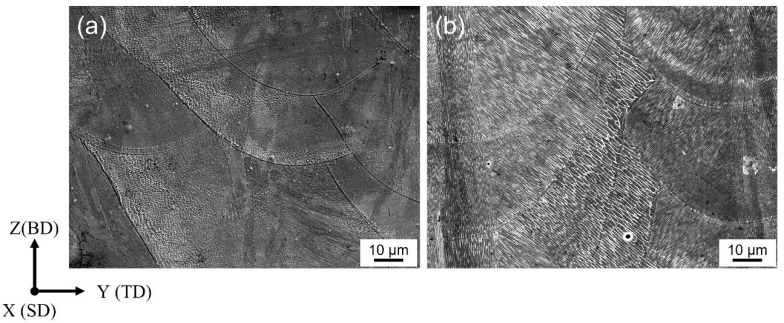
SEM images of the solidification microstructure in (**a**) sample 1 and (**b**) sample 2.

Figure 2a,b show the SEM images of the solidification microstructure in the YZ cross-section of sample 1 and sample 2, respectively, involving melt pool boundaries and columnar structures. Compared with Figure 2a, various white cellular boundaries in Figure 2b can be found clearly due to the slightly different growth orientations. Sample 2 has much more complex cellular structure growth directions than those of sample 1. Cellular structures are formed through epitaxial growth along the preferred crystallographic orientation close to the temperature gradient direction from the molt pool boundaries. The dislocation cellular structure has been proven to grow along the <100> crystallographic direction in alloys with FCC crystals [32]. Local heat flow enables the complex thermal history and various thermal gradient directions. The cellular structures show different morphologies depending on the direction of observation due to the epitaxial growth. Typical dislocation cells appear when the cellular structure growth direction parallels the observation direction. When the cellular structure growth direction is vertical to the observation direction, the longitudinal section of the cellular structure with a lath-like type is observed. When viewed from other arbitrary planes, the actual sizes of the various dislocation cellular patterns can be deduced from the projection geometry. Taking the affecting parameters into consideration, the two samples have the same volumetric laser energy density, which is a function of laser power, laser scan speed, hatch spacing, and layer height. According to Figure 1b, the two samples have the same laser scan speed and layer height and different laser power and hatch spacing. Too much higher laser power may cause vaporization and too much lower laser power may induce insufficient melting of the powder. Laser power has been clarified to be the most influential parameter on the geometrical characteristics of a single track of LPBF-melted stainless steel, followed by other affecting parameters like layer height and laser scan speed [33]. In this case, the laser beam diameter is 0.1 mm during LPBF processing; therefore, the hatch spacing of 0.06 mm in sample 1 and 0.1 mm in sample 2 enable sufficient melting of the powders. Therefore, the solidification structure differences were dominantly affected by the laser power. 

**Figure 3:**
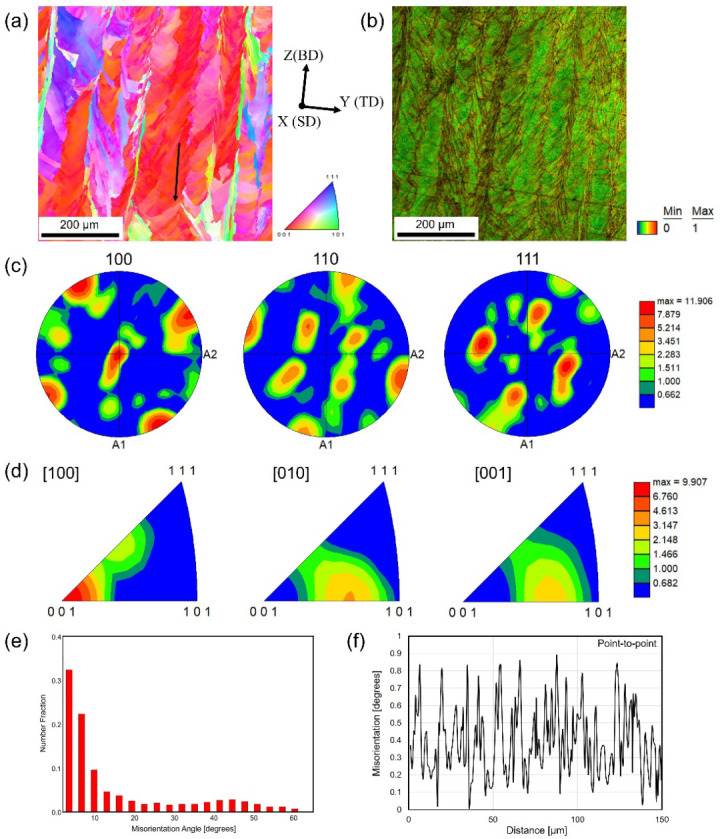
EBSD-measured results of sample 1: (**a**) inverse pole figure (IPF) map plotted along the laser scan direction (SD); (**b**) kernel average misorientation (KAM) map measured in degrees from 0° to 1°; (**c**) pole figures (PF) of crystallographic orientations taken from the YZ plane; (**d**) IPF texture taken from the YZ plane; (**e**) histogram of misorientation angle distribution; (**f**) the point-to-point misorientation along the black arrow shown in (**a**).

Figure 3 shows the crystallographic texture and orientation according to the EBSD data of sample 1 fabricated under P = 187.5 W, v = 1000 mm/s, d = 0.06 mm, t = 0.04 mm, and scan strategy X. Figure 3a shows the IPF map of the YZ cross-section projected in the X direction, acquired with a 1 μm step size. The laser beam size of 100 μm was used during LPBF processing, and the hatch spacing in sample 1 is 0.06 mm. The latter laser track re-melted most of the previous track and solidified again, causing complex microstructure and crystallographic orientations. Some columnar structures with large misorientations can be found clearly, while others with small misorientations were considered directional single crystal-like features. Figure 3b shows the related KAM map, measured in degrees, to illustrate a local misorientation and provides a quantitative analysis of the average misorientation angles. It is evident that most structures have local misorientations in the order of 0–1°. The lamellar structure in the BD-TD plane is displayed clearly in the KAM. Large misorientations existed within the minor layers distributed along BD and arranged parallelly along TD. At the side branching of the minor layers, some misorientations can be clearly found. Combined with Figure 3a, the local regions with large and small misorientations are in one-to-one correspondence. Figure 3c shows the corresponding {100}, {110}, and {111} pole figures. The texture tilts in the YZ cross-section by approximately 25° counterclockwise from the building direction toward the subsequent scan track direction. The IPF maps in Figure 3d show that the preferred orientation of FCC structures along the laser scanning direction (X direction) was the [100] direction with the maximum degree of orientation of 9.907, indicating the anisotropy feature in sample 1. A strong (001) texture with a maximum texture index of 9.907 is present along the SD. Figure 3e shows the number fractions of the misorientation angles from Figure 3a. The grain boundaries with misorientation angles of 2–15° are defined as low-angle grain boundaries while the grain boundaries with misorientation angles larger than 15° are described as high-angle grain boundaries. The grain boundaries with a misorientation of smaller than 2° were not taken into consideration because of the data noise. Therefore, for such a small misorientation measurement in a local region, a misorientation profile along a line is needed to be completed. In Figure 3a, the red local region with single crystal-like columnar structures close to the [100] direction was selected and indicated by the black arrow to take the misorientation profile measurement. Figure 3f shows the point-to-point misorientation profile measurement along the black arrow, indicating a misorientation smaller than 1°, which was consistent with the KAM result as shown in Figure 3b.

**Figure 4:**
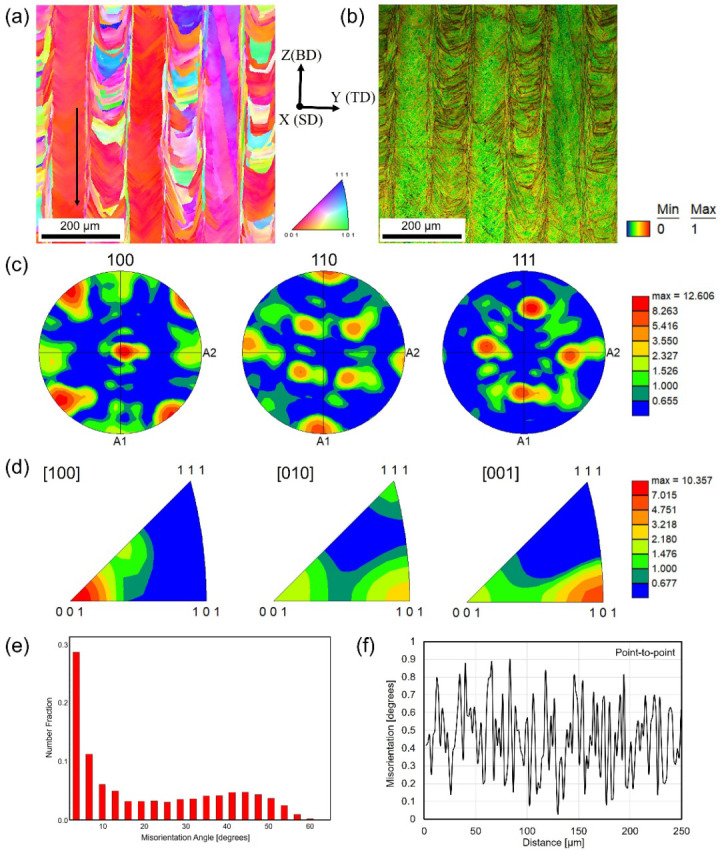
EBSD-measured results of sample 2: (**a**) inverse pole figure (IPF) map plotted along the laser scan direction (SD); (**b**) kernel average misorientation (KAM) map measured in degrees from 0° to 1°; (**c**) pole figures (PF) of crystallographic orientations taken from the YZ plane; (**d**) IPF texture taken from the YZ plane; (**e**) histogram of misorientation angle distribution; (**f**) the point-to-point misorientation along the black arrow shown in (**a**).

Figure 4 shows the crystallographic texture and orientation of sample 2 fabricated under P = 312.5 W, v = 1000 mm/s, d = 0.1 mm, t = 0.04 mm, and scan strategy X. Figure 4a shows the IPF map of the YZ cross-section projected in the X direction, acquired with a 1 μm step size. The hatch spacing in sample 2 is 0.1 mm, equal to the laser beam size. The latter laser track did not re-melt too much of the previous track and solidified again. The lamellar structure with interval distribution behaviors is clearly displayed. The texture and microstructure induced by the different LPBF processing parameters are quite different. Figure 4b shows the related KAM map, measured in degrees, illustrating the local misorientations on the 0–1° order. Similarly, the local regions with large and small misorientations are in one-to-one correspondence with Figure 4a. Figure 4c shows the corresponding {100}, {110}, and {111} pole figures. The texture tilts in the YZ cross-section by approximately 25° counterclockwise from the building direction toward the subsequent scan track direction. The texture of sample 2 (max MUD = 12.606) was slightly stronger than that of sample 1 (max MUD = 11.906). The MUD number is one measure of the texture strength in an EBSD pole figure, with higher values indicating stronger alignment. The IPF maps in Figure 4d show that the preferred orientation of the FCC structures along the laser scanning direction (X direction) was the [100] direction with a maximum degree of orientation of 10.357, which is higher than that of sample 1. In addition, the building direction (Z direction) displayed the preferred orientation along the [001] direction, which is stronger than sample 1. Figure 4e shows the number fractions of the misorientation angle. The grain boundaries with a misorientation smaller than 2° were not taken into consideration. In this case, the number fraction of high-angle grain boundaries with larger than 15° misorientation is higher than that of sample 1, while the number fraction of low-angle grain boundaries with 2–15° misorientation is higher than that of sample 1. To measure the small misorientation in a local region in Figure 4a, the red local region with single crystal-like columnar structures close to the [100] direction was selected and is indicated by the black arrow to take the misorientation profile measurement. Figure 4f shows the point-to-point misorientation profile measurement along the black arrow, indicating a misorientation smaller than 1°, which was consistent with the KAM result as shown in Figure 4b.

**Figure 5:**
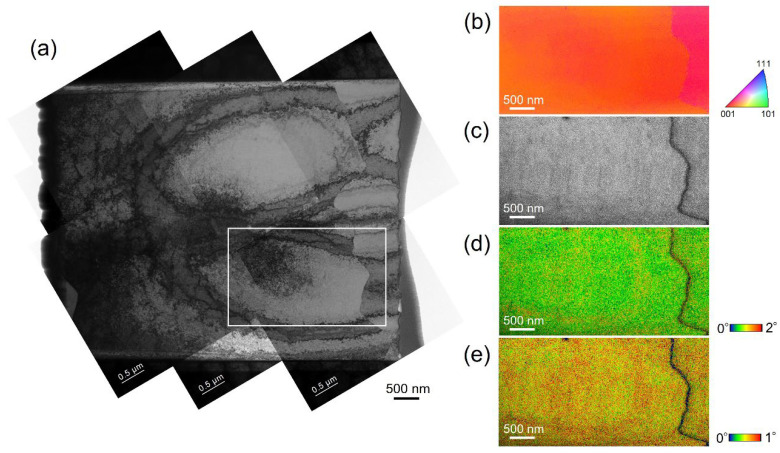
(**a**) TEM bright field image of the dislocation cells along the cross-section of the cellular structure in sample 1; (**b**–**e**) TKD measurement results of the white square region shown in (**a**), including IPF map, IQ map, and KAM map with degree ranges from 0° to 2°, and 0° to 1°, respectively.

It should be noted that the cellular structures found in the SEM images can be observed but not clearly in the IPF and KAM maps according to the conventional EBSD data. The cellular structures have misorientations smaller than 2°, which usually cannot be adequately detected and measured. To further explore and take an exact analysis of the misorientation of smaller than 2° in the adjacent dislocation cells, we used a TKD technique, also known as transmission EBSD (t-EBSD), to obtain the orientation/misorientation information at a higher spatial resolution. Figure 5a shows the TEM image of the cross-section of the cellular structures of sample 1, which is used for t-EBSD analysis with a step size of 30 nm. The TEM specimen was prepared using the FIB method, so the thickness of the specimen decreases gradually from the left side to the right side. Dislocation cells were found clearly in the relatively thick region, while they were invisible in the very thin region. However, the very thin region is suitable for TKD measurement, so the region indicated by the white square was selected for TKD measurement. Figure 5b shows the IPF map acquired from the t-EBSD data. The grain boundary on the right side was demonstrated, while the dislocation cells cannot be detected clearly. Figure 5c shows the related image quality (IQ) map. The dislocation cells remained invisible. Figure 5d,e show the KAM maps with the degree range from 0 to 1° and 0 to 2°, respectively. A large number fraction of the misorientation smaller than 1° can be identified, even though the dislocation cell boundaries were not detected. The t-EBSD analysis results were consistent with the above-mentioned results and further confirmed the existence of the misorientation smaller than 1°.

**Figure 6:**
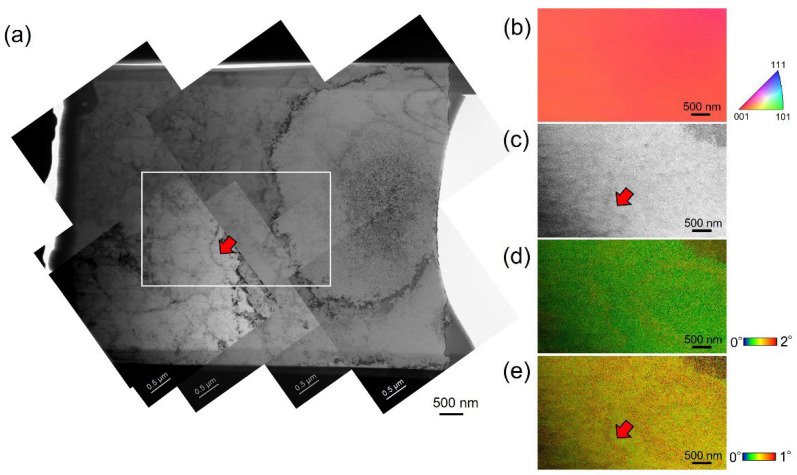
(**a**) TEM bright field image of the dislocation cells along the cross-section of the cellular structure in sample 2; (**b**–**e**) TKD measurement results of the white square region shown in (**a**), including IPF map, IQ map, and KAM map with degree ranges from 0° to 2°, and 0° to 1°, respectively. Red arrows in (**a**,**c**,**e**) indicate the same cell boundary.

In the case of sample 2, based on the analysis results of sample 1, the local region for t-EBSD analysis was selected at a little bit thicker region. Here, the trade-off between in-plane resolution and slice thickness could potentially affect the delineation of the cell structures. The affecting factors may also consist of the surface quality of the TEM specimen, scanning step size, and so on. Figure 6a shows the TEM bright field image of the specimen of sample 2 for the TKD measurement. Similarly, the region indicated by the white square was selected for TKD measurement. In this case, the red arrow indicates the cell boundary, which is expected to be detected during the TKD analysis. Figure 6b shows the IPF map, displaying no obvious misorientation. In the IQ map, shown in Figure 6c, the red arrow indicates the cell boundary, which is the same as in Figure 6a. Some slight traces on the left side of the IQ map may display the cell boundaries. Figure 5d,e show the KAM maps with the degree range from 0 to 2° and 0 to 1°, respectively. Still, a large fraction of the misorientation smaller than 1° can be identified. In Figure 5e, the red arrow indicates the clear misorientation of the cell boundary from the one shown in Figure 5a. The t-EBSD analysis method is also available for analysis of the small misorientation. 

**Figure 7:**
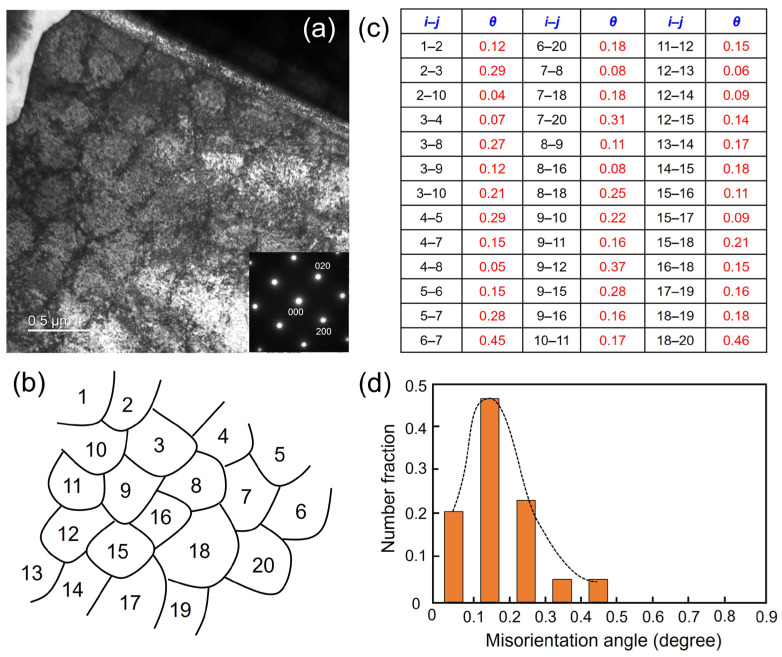
(**a**) TEM bright field image of dislocation cells and related diffraction pattern of sample 1; (**b**) schematic diagram of the dislocation cells with indexing from 1 to 20; (**c**) calculated misorientation angle *θ* between adjacent cells *i* and *j*; (**d**) histogram of misorientation angle distribution.

To continue analyzing the misorientation between the adjacent dislocation cells, the same TEM specimens of TKD measurement were performed to obtain the Kikuchi pattern from each dislocation cell for further calculation. Figure 7a shows the TEM bright field image of the dislocation cells from the upper-left region in Figure 5a. The inset diffraction pattern from the whole specimen indicates the single crystal-like structure along the [001] direction. The dislocation cells with an average size of 400 nm can be observed. Figure 7b shows the schematic diagram of the dislocation cells with numbers from 1 to 20. After obtaining the Kikuchi pattern, the misorientation between two adjacent dislocation cells was calculated using TOCA software. Figure 7c lists all the misorientations between each dislocation cell. Figure 7d shows the misorientation angle and related number fractions of these dislocation cells. All 20 dislocation cells analyzed have misorientation angles smaller than 0.5°. The misorientation angles ranging from 0.1 to 0.2° account for the largest proportion. The accurate and quantitative TEM analysis results give much more detail.

**Figure 8:**
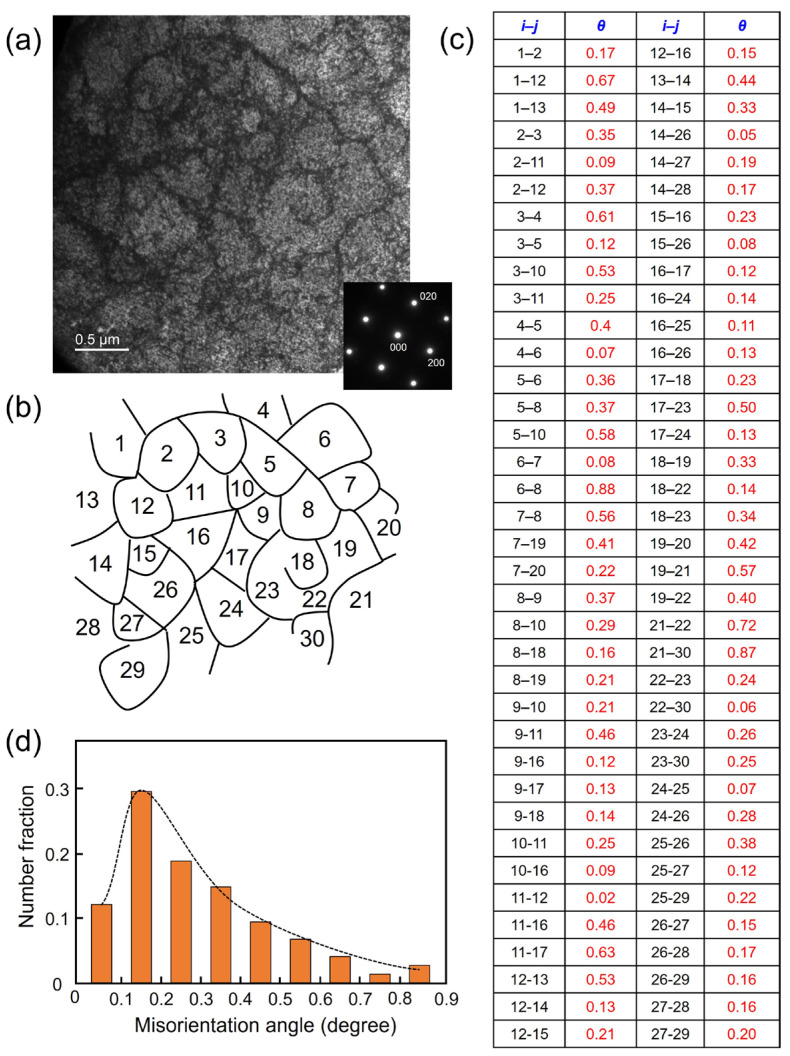
(**a**) TEM bright field image of dislocation cells and related diffraction pattern of sample 2; (**b**) schematic diagram of the dislocation cells with indexing from 1 to 30; (**c**) calculated misorientation angles *θ* between adjacent cells *i* and *j*; (**d**) histogram of misorientation angle distribution.

Figure 8a shows the TEM bright field image of the dislocation cells from the lower-left region in Figure 6a. The inset diffraction pattern from the whole specimen indicates the single crystal-like structure along the [001] direction. The dislocation cells with an average size of 400 nm can also be observed, which is similar to that of sample 1 without a big difference. Figure 8b shows the schematic diagram of the dislocation cells with numbers from 1 to 30. Figure 8c lists all the misorientations between each dislocation cell. Figure 8d shows the misorientation angle and related number fractions of these dislocation cells. All 20 analyzed dislocation cells have misorientation angles ranging from 0° to 0.9°. The misorientation angles ranging from 0.1 to 0.2° also account for the largest proportion. 

Generally, the formation of the solidification cellular structure is governed by the temperature gradient (G) and the crystal growth rate (R) within the melt pool of the alloys during LPBF. It has been identified that the G/R ratio plays a crucial role in the determination of solidification morphology, and the high G/R ratio induced by high cooling rate of LPBF could effectively produce a group of cylindrical cells having a cross-section of honeycomb shape growing along the G direction from the MPBs [26,34]. Due to the variation in the heat flow condition and the Benard–Marangoni instability in front of the solid/liquid interface inside the melt pools, the cell morphologies could be locally varied within a melt pool, and the corresponding misorientation between adjacent cells could also be different. The competition between epitaxy, preferential growth direction, and temperature gradient largely determines the final grain orientation. While small misorientations may not significantly compromise the mechanical properties or functionality of the printed part, they can affect its overall quality and performance. In addition, minimizing small misorientations in cellular structures during solidification requires careful control of the process parameters, including temperature gradients, cooling rates, and melt pool dynamics. Advanced process monitoring techniques, such as in situ sensing and thermal imaging, can help identify and mitigate potential sources of misorientation during AM solidification. Additionally, optimization of build strategies and post-processing treatments can improve the overall quality and integrity of AM-produced components.

## 4. Conclusions

This study analyzed the misorientation angles between cellular structures in LPBF-processed 316L SS, mainly ranging from 0 to 2° using conventional EBSD, TKD, and TEM techniques. The conventional EBSD technique presents the misorientations in the IPF map between the cellular structures without consideration of the angle smaller than 2°. The KAM map and misorientation analysis of point-to-point could display the misorientations smaller than 1°. The TKD analysis results with higher in-plane resolution still cannot display the misorientations between the adjacent dislocation cells and also present misorientations smaller than 1°. The affecting factors may include surface quality and thickness of the TEM specimen, scanning step size, and so on. TEM analysis results show the accurate misorientation angles between adjacent dislocation cells according to the Kikuchi pattern acquired from each dislocation cell. The misorientation angles have been identified with the range from 0 to 0.5° in sample 1 and 0 to 0.9° in sample 2. The misorientation angles range from 0.1 to 0.2° also account for the largest proportion in both samples. The two samples have the same volumetric laser energy density, same laser scan speed and layer height, and different laser power and hatch spacing. The competition between epitaxy, preferential growth direction, and temperature gradient largely determines the final grain orientation. The cellular boundary with dislocations balances the solidification strain and residual stresses among neighboring cellular structures. This study presents that the TEM method is the better and more accurate analysis method of the measurement of the cellular structures and provides insights into measuring the small misorientation angle between adjacent dislocation cells and nanograins in nanostructured metals and alloys with ultrafine-grained microstructures.

## Data Availability

Data are contained within the article.
